# Cardiac and vascular complications in lupus: Is there a role for sex?

**DOI:** 10.3389/fimmu.2023.1098383

**Published:** 2023-03-29

**Authors:** Alexa Corker, Maya Learmonth, David M. Patrick, Kristine Y. DeLeon-Pennell, Justin P. Van Beusecum

**Affiliations:** ^1^ Division of Cardiology, Department of Medicine, Medical University of South Carolina, Charleston, SC, United States; ^2^ Division of Clinical Pharmacology, Department of Medicine, Vanderbilt University Medical Center, Nashville, TN, United States; ^3^ Department of Research Service, Tennessee Valley Healthcare Veterans Affairs (VA) Medical Center, Nashville, TN, United States; ^4^ Department of Research Service, Ralph H. Johnson Veterans Affairs (VA) Healthcare System, Charleston, SC, United States; ^5^ Division of Nephrology, Department of Medicine, Medical University of South Carolina, Charleston, SC, United States

**Keywords:** lupus, sex differences, cardiovascular complications, vascular, cardiac

## Abstract

Systemic lupus erythematosus (SLE) is a common systemic autoimmune disorder and is characterized by autoantibody formation and subsequent immune complex deposition into target organs. SLE affects nearly nine women to every one man worldwide. Patients with SLE are at an enhanced risk for cardiovascular disease (CVD) morbidity and mortality. CVD is the leading cause of death worldwide and includes heart and blood vessel disorders, cerebrovascular disease, and rheumatic heart disease. Specific mechanisms by which cardiac and vascular pathophysiology develops in patients with SLE are still not fully known. Not only do we not understand this correlation between SLE and CVD, but there is also a critical gap in scientific knowledge on the contribution of sex. In this review, we will discuss the cardiac and vascular pathological disease states that are present in some patients with SLE. More importantly, we will discuss the potential mechanisms for the role of sex and sex hormones in the development of CVD with SLE.

## Introduction

While overall mortality due to complications arising from patients with systemic lupus erythematosus (SLE) has decreased with the development of new therapies in recent years, the risk of mortality from cardiovascular disease (CVD) in patients with SLE has remained steady over time (1). Patients with SLE have a 3-4 times higher risk of experiencing a CVD-associated event and mortality from these events compared with patients without SLE (2). This includes accelerated risks for hypertension, vascular dysfunction, and myocardial infarction (MI) (3, 4). Clinically, SLE predominantly affects women more than men (women *versus* men ratio of 9 to 1), limiting the availability of data on male SLE patients (5). Nonetheless, both male and female patients with SLE have an increased risk for CVD-related mortality (6). Studies that focus solely on men with SLE and related comorbidities are even rarer and only look at symptom prevalence and commonalities between patients with SLE within the study group (7). Despite mechanistic research being limited in men with SLE, case studies can potentially shed insight into the complexity of SLE diagnosis and CVD complications (5). This review will highlight the current knowledge of sex differences in SLE and describe potential mechanisms. To determine the pathophysiology of vascular and cardiac complications in SLE, we will also review the research gap in sexual dysmorphisms seen in CVD complications in SLE. Research into the mechanistic, symptomatic, and immunological differences between male and female patients with SLE and CVD complications may be able to highlight new therapies to lower this associated risk.

## Sex differences in the pathogenesis of SLE

**Figure 1:**
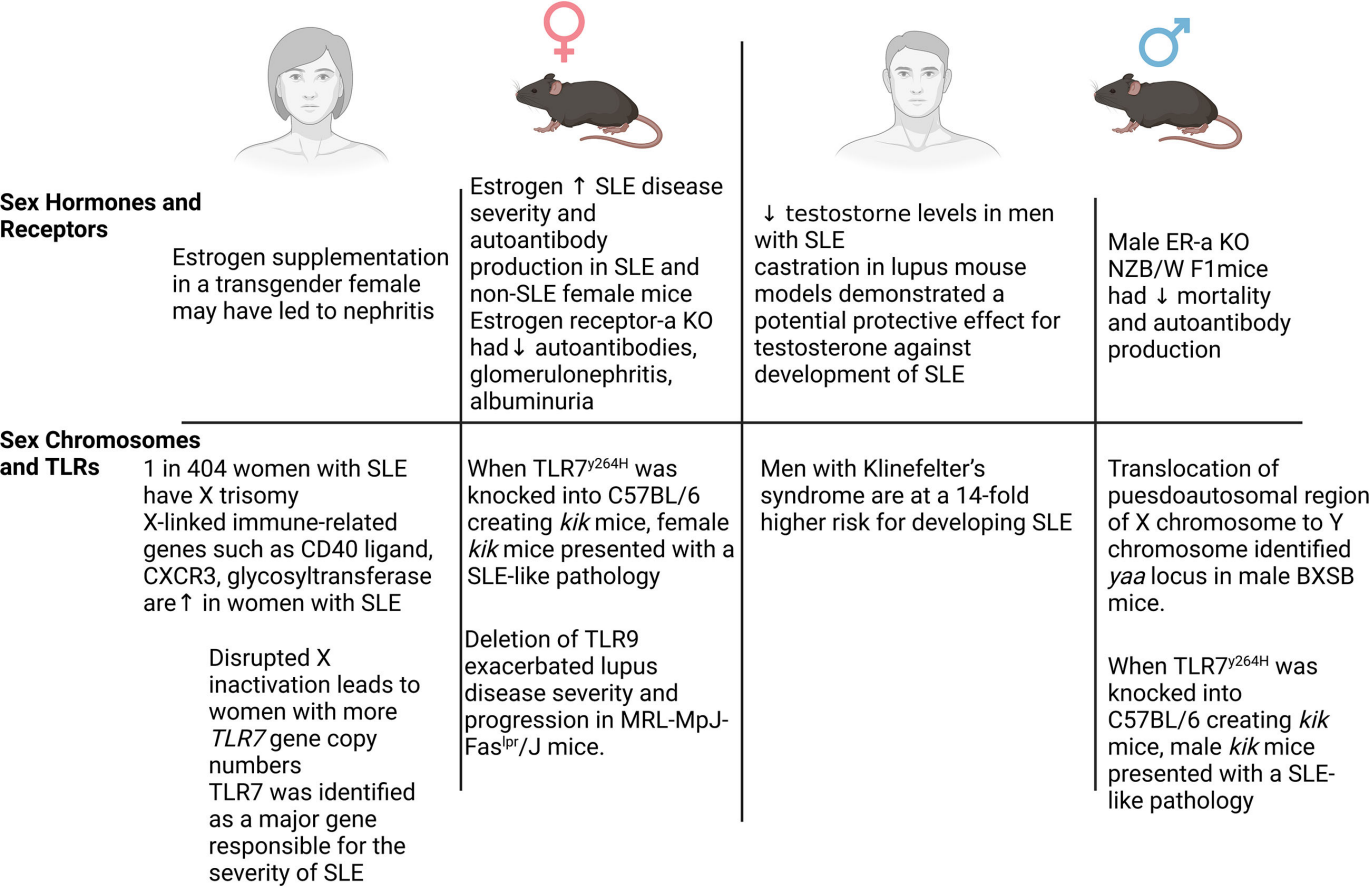
Sex differences in systemic lupus erythematosus (SLE). Numerous mechanisms have been demonstrated to contribute to the sex-dependent development of SLE in both patients and animal models. Sex hormones and receptors, sex chromosomes, and Toll-like receptors all have been implicated as key mechanisms in SLE. In female patients and mice (left), elevated estrogen and estrogen receptor signaling, along with the X chromosome-linked proinflammatory genes (*Cxcr3*, *Cd40*, *Tlr7*), contribute to the frequency and/or severity of SLE. In male patients and mice (right), an imbalance of estrogen and testosterone, elevated estrogen receptor signaling, increased copies of the X chromosome (Klinefelter’s syndrome), and TLR7 signaling contribute to the severity and development of SLE.

SLE affects multiple organs and is extremely heterogeneous in its clinical presentation across different patients. Like many other autoimmune diseases, SLE affects women more frequently than men; however, male patients with SLE tend to have a more rapid and severe disease state (8–10). This puzzling observation has led numerous studies to investigate sex differences in the development and pathogenesis of patients with SLE. Male patients with SLE experience a higher occurrence of renal involvement than women; however, progression to end-stage renal disease is not different between sex (8–10). While there are numerous mechanisms that contribute to sex differences in SLE development between male and female patients, we will discuss the role of sex hormones and chromosome complement below (**Figure 1**).

### Sex hormones and receptors

Diagnosis of SLE in women occurs usually when estrogen is at peak levels during a patient’s lifetime, leading one to assume that sex hormones contribute to the pathogenesis of SLE. In line with this, in a common lupus mouse model, the New Zealand black/white (NZBW F1), ovariectomy of female NZB/W F1s delays the onset of autoantibody production, suggesting the important role of female sex hormones including estrogen (11). Interestingly, in the MRL/Lpr mouse model, which affects male and female mice equally, female mice develop earlier and a more severe case of SLE compared with male mice (12). However, a clinical study by Hill et al. demonstrated that estrogen supplementation in transgender women may have resulted in lupus nephritis (13). This clinical study mimics the findings in the NZB/W F1 mouse model as treatment with androgens suppressed the development of autoimmune disease and improved overall mortality in female mice, indicating that the balance between sex hormones may play a critical role in SLE pathogenesis (14). In a non-autoimmune prone mouse (C57BL/6), estrogen treatment increased the rate of nephritis and autoantibody production, suggesting that estrogen may play a critical role through modulation of the immune system (15, 16).

While estrogen has been implicated in contributing to the development of SLE, numerous impactful studies have shed light on the role of estrogen receptors. The pioneering study showing evidence of estrogen receptor signaling in lupus comes from Bynote et al. who used the estrogen receptor-α knockout (KO) on the NZB/W F1 background (17). In this study, ER-α KO NZB/W F1 female mice had reduced development of autoantibodies, glomerulonephritis, and albuminuria and increased survival time compared with controls. Similarly, male ER-α KO NZB/W F1 mice had decreased mortality and a reduction in autoantibody production (17). In keeping with this, ER-α KO mice on the NZM2410 and B6SLE1b backgrounds exhibited reduced glomerulonephritis and proteinuria and increased survival, likely through a reduction in B- and T-cell hyperactivation (18, 19). While ER-α KO mice have provided critical evidence on the role of estrogen in the development of SLE, an ER-β KO on a lupus background has not yet been developed. Despite the lack of genetic KO, in ovariectomized female NZW/B F1 mice activated with a selective ER-β agonist, anti-DNA autoantibodies were reduced; however, albuminuria and survival were not affected (20). Together, these studies suggest that ER-α plays a predominant role in the sex-dependent development of SLE.

Men with SLE have been shown to have decreased serum testosterone levels compared with healthy controls (21). Whether this decrease in testosterone was a predisposition for SLE disease onset or if the decrease was due to chronic SLE disease was not investigated. Studies using castration in lupus mouse models demonstrated a potential protective effect of testosterone against the development of SLE (22). Despite being performed in women, clinical observational studies further corroborated the protective effects of androgens in SLE patients who displayed decreased disease burden through testosterone therapy (23, 24).

### Sex chromosomes

While sex hormones have been attributed a partial role in the development of SLE, both prepubescent and postmenopausal women can develop SLE, suggesting that an alternative mechanism independent of sex hormones is also responsible for SLE pathogenesis. Sex chromosomes have been implicated to contribute to the development of SLE in both sexes. Importantly, the frequency at which rare X chromosome abnormalities occur in patients with SLE is much higher than in the general healthy population (25). Approximately 1 in 404 women with SLE has X trisomy (47, XXX), while men with Klinefelter’s syndrome (47, XXY) are at an approximately 14-fold higher risk for developing SLE (26, 27). Not only are X chromosome abnormalities linked to SLE development in male and female patients, but also disturbed X chromosome inactivation contributes to the pathogenesis of female patients with SLE. Numerous immune-related genes are contained in the X chromosome. Hewagama et al. demonstrated that X-linked immune-related genes such as the CD40 ligand, CXCR3, and glycosyltransferase (OGT) are upregulated in women with SLE (28). Interestingly, this study demonstrated that X chromosome demethylation in women played a role in the predisposition to lupus. This finding has been expanded further with additional studies demonstrating that SLE patients have significant reductions in epigenetic modifications on the inactive X and aberrant X-linked gene expression leading to abnormal autoantibody production, increased expression of type I interferon (IFN)-regulated genes, and upregulation of immune-regulatory genes on T cells (29–31).

Toll-like receptor (TLR) 7 and TLR9 are expressed on the X chromosome and have been demonstrated as potential mechanisms for the development of SLE providing an additional mechanism for promoting an inflammatory response (32). Numerous studies demonstrated that sex differences in TLR7 signaling may be due to disrupted X inactivation (46, XX) leaving women with more *TLR7* gene copy numbers (32–34). This was demonstrated by the translocation of a segment near the pseudoautosomal region of the X chromosome onto the Y chromosome identifying the y-linked autoimmune accelerating locus (*yaa*) in male BXSB lupus mice (35). Among the key genes that were duplicated in the *yaa* locus, *TLR7* was identified as a major gene responsible for the severity of SLE (35). Recently, Brown et al. demonstrated that both male and female mice presented with an SLE-like pathology using a human TLR7 gain-of-function mutation (TLR7^y264H^) mouse model. TLR7^y264H^ was knocked into C57BL/6 creating *kik* mice (36). TLR7 is not the only TLR that has been implicated in the development of SLE; however, the findings are paradoxical and inconclusive. For instance, despite a positive correlation with SLE incidence, TLR9 deletion in lupus-prone mice did not reduce the severity of the disease. Moreover, TLR9 deletion led to an exacerbation of lupus in mouse models (37). In contrast, Cunningham et al. demonstrated that ER-α binding to the estrogen-responsive element led to the production of proinflammatory cytokines from TLR7/8 and TLR9 activation, suggesting that sex hormones and chromosomes provide a synchronous mechanism that likely regulates SLE pathology (38). Together, these studies suggest that X chromosome abnormalities in SLE patients likely are driving the reactivation of numerous immune-related genes and the promotion of autoimmunity.

## Mechanisms of CVD complications in SLE

**Figure 2:**
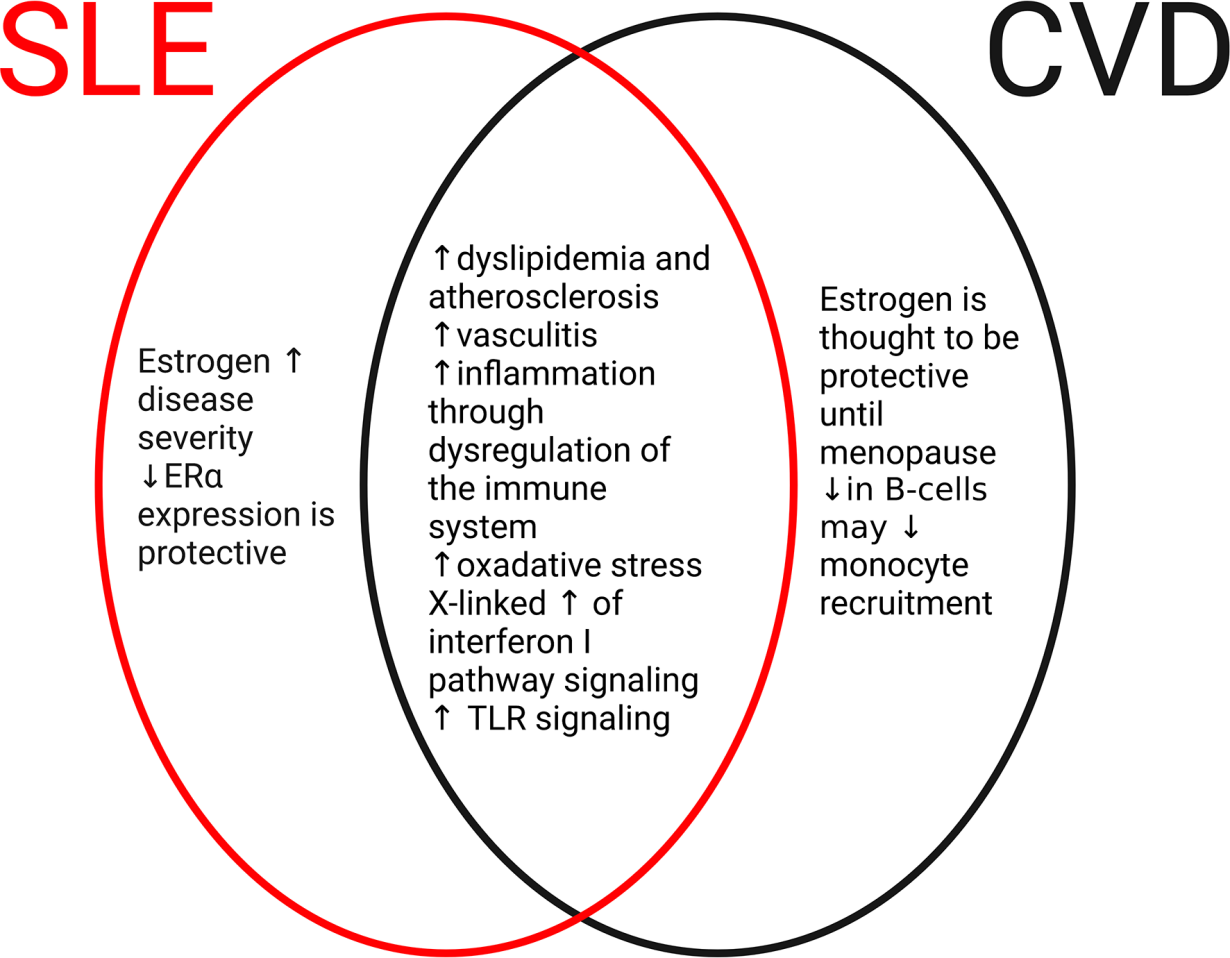
Common and disparate mechanisms of SLE and cardiovascular disease (CVD). In the development of SLE or CVD, there are both disparate and common mechanisms. In SLE (red oval), elevated levels of estrogen and estrogen receptor signaling promote the severity and frequency of the disease. In CVD (black oval), estrogen and receptor signaling are cardioprotective until menopause. Differences in immune cell populations including B cells and monocytes may contribute to CVD. However, numerous mechanisms are shared between SLE and CVD (middle oval) including dysregulation of the immune system, increases in oxidative stress, and development of atherosclerosis through dyslipidemia. These common pathways may contribute to CVD complications that are frequently seen in patients with SLE.

It is well established that an SLE diagnosis is linked to an increased risk of CVD including atherosclerosis, coronary artery disease, cerebrovascular events, myocardial infarction, and peripheral artery disease (39–41). Compared with patients without SLE diagnosis, female patients with SLE are at risk approximately 3-4 times for experiencing a CVD event including cardiac complications (2, 6, 39). In this section, we will briefly discuss the cardiac and vascular complications that can occur in SLE (**Figure 2**).

### Vascular

Nearly 10% of all patients with SLE have accelerated atherosclerosis development; inflammation in small, medium, and large vessels; and impaired endothelium-dependent relaxation compared with age-matched controls (3, 42–46). Multiple clinical studies implicate dyslipidemia and clotting factors in the association between CVD and SLE (5, 43, 47). In a study published by Urowitz et al., both male and female patients with SLE had a 60% increased prevalence of dyslipidemia 3 years after study enrollment (48). In another study, young women with juvenile SLE had decreased HDL and elevated VLDL levels compared with healthy girls reaching limits comparable to age-matched healthy boy controls (49). These data indicated that SLE can shift the lipid profile of young women which may predispose them to premature CVD development, which would not otherwise be seen in healthy age-matched women (49). The opposite trend was found in young men with juvenile SLE who had elevated HDL subsets and decreased VLDL subsets compared with age-matched healthy controls. This shift in lipid profile in young men with juvenile SLE may be due to low testosterone levels that has been observed in male SLE patients (21, 50). These data present that sex differences in lipid profiles in juvenile SLE patients, along with chronic inflammation associated with juvenile SLE, may influence the risk of vascular disease.

The lifetime prevalence of vasculitis in SLE patients is estimated to be as high as 56% with large studies showing a prevalence of 11% to 36% (51, 52). The consequences of vasculitis range from cutaneous manifestations to life-threatening organ dysfunction. Interestingly, the territories typically associated with lupus vasculitis include cutaneous vessels, renal small vessels, coronary arteries, and vessels of the brain; however, pulmonary and gastrointestinal involvement is less common (53). Clinically, it is important to separate inflammatory vasculitis from antiphospholipid syndrome which may present with similar manifestations. Vasculitis in SLE is characterized by inflammatory cell infiltration into the vessel that is initiated by the formation or deposition of immune complexes into the vessel wall (51). Interestingly, patients with vasculitis are younger and primarily male, suggesting the importance of biological sex in the pathogenesis of SLE-associated vasculitis (51). While the complete mechanisms of the development of vascular dysfunction and inflammation in SLE are unknown, both basic research and clinical studies have identified several key contributors.

Similarly, anticardiolipin antibodies and antiphospholipid syndrome have been observed in SLE patients (between 30% and 40%) and are known risk factors for clotting disorders (47). While there have not been many studies evaluating sex differences, a cohort study conducted by Bayraktar et. al., investigating the clinical spectrum of catastrophic antiphospholipid syndrome in patients with and without SLE, demonstrated that at the time of disease presentation, patients with SLE were more likely to be female and younger, have cerebral and pancreatic involvement, receive corticosteroids and cyclophosphamide, and have a higher risk of mortality after adjusting for age, sex, organ involvement, and treatment than those without SLE (54). In a separate study, greater than 55% of SLE patients presented with antiphospholipid antibodies and organ damage at a 15-year follow-up. Interestingly, in this study, older men were associated with an increased risk of organ damage, in contrast to the findings of Bayraktar et al. It is likely that these observed differences were due to differences in disease stage and possibly due to the sample sizes of men *versus* women (55). Importantly, these key contributors are mostly demonstrated in female patients, and future studies including male patients with SLE are needed to investigate true sex-dependent mechanisms.

### Cardiac

Cardiac-related mortality is significantly increased in both male and female patients with SLE than in non-SLE patients (6). A commonality between cardiac disease and SLE is a dysregulation of the immune system. This has been demonstrated by numerous groups where aberrant antigen-presenting cells (macrophages, dendritic cells, and B cells) and T-cell activation promote both CVD complications and SLE disease progression (56–59). Increased inflammatory activity and oxidative stress in SLE patients are associated with an increased risk for cardio-specific pathologies including myocarditis, atherosclerosis, and MI (40, 60). Whether this dysregulation is due to overreactive immune cells or sex hormone levels is unknown, both of which may be implicated in SLE pathogenesis.

While SLE may not affect the observed sex differences in the type of vascular obstruction, premature atherosclerosis is a severe risk factor for SLE patients (61, 62). Women with lupus between 35 and 44 years old were over 50 times more likely to have an MI than women of similar age in the Framingham Offspring Study (rate ratio = 52.43, 95% CI: 21.6-98.5) (62). With advancements in treatment and increased life expectancy of SLE patients, CVD has become a more prevalent health issue in SLE patients. More recently, Tornvall et al. investigated 4,192 patients with SLE and 41,892 non-SLE age-matched control patients (83.2% women in each cohort). During the 20-year follow-up, the incidence of MI was 9.6 (95% CI: 8.9–10.5) and 4.9 (95% CI:4.8-5.1) events/1,000 person-years in patients with SLE and controls, respectively (63). Even still, the impact of this problem has been understudied and underrecognized. Future studies should focus on the aggressive management of hypercholesterolemia and other possible risk factors for plaque formation. In addition, early detection of atherosclerosis may provide an opportunity for therapeutic intervention.

## The role of sex hormones and chromosomes in CVD complications in SLE

### Sex hormones

While we acknowledge that sex-dependent mechanisms in the pathogenesis of SLE are more complex than these key mechanisms, similar mechanisms have been implicated in CVD alone and SLE alone. Sex hormones have been implicated to influence CVD prevalence and prognosis through multiple mechanisms including their effects on the immune system. Serum from SLE patients with higher levels of estrogen positively correlates to SLE disease severity (64). In non-SLE patients, a high estrogen:testosterone ratio has been considered cardioprotective; however, due to the findings from the Women’s Health Initiative (WHI), hormone therapy has been demonstrated to have a complex pattern of risks and benefits with younger women having a greater benefit than older women (age 70 to 79), who had a trend toward increased risk for adverse CVD events (65–71). While there is a clear link between estrogen signaling and SLE with the loss of ER-α being protective, the role of estrogen in CVD progression in SLE patients is not as clear. Estrogen signaling has been shown to be attenuated with age in animal models of hypertension and myocarditis likely due to a decrease in ER-α levels which has been shown to stimulate vasoconstriction and modulate immune cell activation highlighting a divergent mechanism compared with SLE literature (72, 73).

One potential mechanism for estrogen loss of protection in SLE *versus* non-SLE settings may be due to the cell type most affected in these two disease states as patients with SLE are more likely to have B-cell dysregulation which is not as common when evaluating non-SLE patients. Estrogen signaling *via* ER-α acts in a B-cell intrinsic manner to promote the development of autoantibodies (74). Taking what we know from the SLE studies, we can extrapolate that the adverse effects of estrogen *via* ER-α in the setting of CVD may be due to an exacerbation of the immune response which is a known critical player in multiple cardiovascular pathologies. Future studies further evaluating the commonalities in addition to the differences between these two disease settings may provide additional evidence for the increased risk of CVD in patients with SLE.

Male sex is a traditional risk factor for CVD, and with SLE, there has been some indication that there is a compounded risk of CVD in men. Whether this is due to sex hormones or some other mechanisms is unknown. The literature on testosterone and the regulation of inflammation is mixed with some studies indicating testosterone has anti-inflammatory properties while others indicate the opposite (75, 76). Early studies have shown that a decrease in testosterone increased circulating inflammatory cytokine expression and the readdition of testosterone protected human and mouse models that demonstrate deficiency (77). Due to the known correlation between prolonged or exacerbated immune response and CVD, it is reasonable to conclude that altered testosterone levels in men with SLE may be facilitating poor prognosis after CVD incidence.

Sex hormones, more specifically testosterone, have been found to influence dyslipidemia which is a risk factor for CVD. In a retrospective cross-sectional study including 442 men and 2,122 women, dyslipidemia prevalence was increased in men and women with SLE and increased testosterone (78). In men, the prevalence of dyslipidemia and testosterone levels were dependent on age, whereas dyslipidemia in women and the effect of testosterone were dependent on menopausal status (78). There is a need for further investigation of the role of sex hormones and how they contribute to CVD complications in patients with SLE and in mouse models of SLE.

### Sex chromosomes

To evaluate the effects of sex chromosomes in CVD pathology, the most common animal model used is the four-core genotypes (FCG) mouse model. To generate the FCG mouse, the testis-determining gene *Sry* is removed and replaced on an autosome resulting in a gonadal type model not controlled by the sex chromosomes (79). Using this model, the X chromosome has been implicated in promoting injury due to elevated expression of inflammatory genes including the CD40 ligand, interleukin-1 receptor-associated kinase 1, and FoxP3 along with genes that promote apoptosis, lipid oxidation, and generation of oxygen-derived free radicals (33, 80, 81). These findings are similar to what has been observed in SLE patients which, as discussed above, have demonstrated that upregulation of X-linked genes dysregulates the immune response. This loss in the balance regulating the immune response in SLE patients would likely exacerbate the response to CVD, further promoting disease pathology. The X-linked upregulation of interferon I pathway signaling associated with SLE patients has also been linked to poor atherosclerosis outcomes through potentiation of foam cells, extracellular traps, endothelial dysfunction, and pro-injurious dendritic cell phenotypes (82–84). Interestingly, self-DNA released from either dying cells or neutrophil extracellular traps in atherosclerotic lesions has been hypothesized to stimulate autoimmune activation of dendritic cells aggravating atherosclerosis lesion formation, implicating a potential overlapping mechanism between SLE and atherosclerosis (83). In the case of MI in the non-SLE setting, B-cell depletion has been shown to be beneficial due to an attenuation of monocyte recruitment *via* Toll-like receptor activation and B-cell secretion of Ccl7 (85). Autoantibody formation against myocardial epitopes has not yet been studied in this context; however, it is likely that SLE would play a big role in the post-MI response.

There have been numerous TLRs implicated in the development of CVD alone especially TLR2, TLR4, and TLR9 (38, 86, 87). Interestingly, there is very little known about the role of TLRs in the development of CVD complications in SLE, even though TLRs play an important role in the pathogenesis of SLE. Recently, Elshikha et al. reported that TLR7 activation with resiquimod (R848) promoted the development of CVD complications in *B6SLE123* mice (88). After TLR7 agonism with R848, *B6SLE123* mice developed a CVD pathology including microvascular inflammation and myocytolysis of the heart (88). CD45^+^ leukocyte infiltration into the heart was higher in female mice compared with males; however, no other sex differences were further investigated (88). Without full evaluation, this could lead the scientific community to assume that there were no sex differences in TLR7 agonism in *B6SLE123* mice in the development of CVD complications highlighting the need for clear transparency when presenting findings. Although a direct investigation of sex differences in CVD complications in SLE was not conducted, there are clear associations between TLR signaling, specifically TLR7 and TLR9, in the development of both SLE and CVD independently. Future studies need to be conducted in both sexes.

## Future directions

Current data suggest that men with SLE have a higher predisposition and more severe disease presentation of CVD compared with women with SLE; however, the mechanisms behind this remain unclear (10, 89, 90). As CVD is the leading cause of death worldwide, there is critical importance to address this large gap in scientific knowledge and begin to unwind the key players that link these two disease states (**Figure 2**). One critical question that remains unanswered is are the drivers of cardiac complications without concomitant SLE the same or different than those with concomitant SLE? As discussed in the sections above, SLE disease severity in both men and women is linked to a higher risk of negative cardiovascular outcomes with one potential mechanism being chronic inflammation. Under non-SLE settings, studies have indicated that despite similarities in the pathological endpoint, the pathway and signaling mechanisms that stimulate disease progression differ between the sexes (91). Whether or not this is true in SLE patients is not known; however, it can be assumed that due to sex differences in SLE pathology, the pathway to CVD progression also likely differs. Interrogating signaling mechanisms that may differ in men *versus* women with SLE could provide invaluable information on patient-specific therapeutics. Answering this fundamental question from a basic science and translational research standpoint could have vast clinical implications for the future of patients with CVD complications and SLE.

## Conclusion

Despite SLE being associated with a significantly increased risk for the development of CVD, there is still much to be learned regarding the mechanisms that drive SLE contributions in CVD pathology. There is limited clinical, translational, and even fewer basic science research investigating sex differences in CVD complications with SLE. Moreover, much of the current literature only includes women with SLE and female mouse models, providing little to no insight into the sex-dependent mechanisms of CVD complications in SLE. There is a clear scientific and clinical gap in the understanding of the physiological and pathophysiological mechanisms of the development of CVD complications in men with SLE. Future studies should focus on characterizing sex difference-specific mechanisms in the development of cardiac and vascular complications in SLE. It is also imperative that clinical studies include male patients with SLE to begin to investigate the role of sex and sex hormones as biological variables in CVD complications in SLE. These approaches may lead to a better understanding of this complex disease process which help identify potential preventatives or therapeutic treatments to reduce or eliminate CVD morbidity and mortality in SLE.

## Author contributions

AC and JPVB prepared the figures. AC, ML, DMP, KYD-P, and JPVB drafted the manuscript. AC, KYD-P, and JB edited the manuscript. AC, ML, DMP, KYD-P, and JPVB approved the final version of the manuscript. All authors contributed to the article and approved the submitted version.
